# Diet induced insulin resistance is due to induction of PTEN expression

**DOI:** 10.21203/rs.3.rs-4021885/v1

**Published:** 2024-06-27

**Authors:** Neal Rosen, Radha Mukherjee, Priya Pancholi, Malvika Sharma, Hilla Solomon, Merna Timaul, Claire Thant, Rory McGriskin, Omar Hayatt, Vladimir Markov, John D’Allara, Simona Bekker, Jacqueline Candelier, Sebastian Carrasco, Elisa de Stanchina, Kiran Vanaja

**Affiliations:** Memorial Sloan Kettering Cancer Center; Memorial Sloan Kettering Cancer Center; Memorial Sloan Kettering Cancer Center; Memorial Sloan Kettering Cancer Center; Memorial Sloan Kettering Cancer Center; Memorial Sloan Kettering Cancer Center; Memorial Sloan Kettering Cancer Center; Memorial Sloan Kettering Cancer Center; Memorial Sloan Kettering Cancer Center; Memorial Sloan Kettering Cancer Center; Memorial Sloan Kettering Cancer Center; Weill Cornell Medicine; Memorial Sloan Kettering Cancer Center; MSKCC; Memorial Sloan Kettering Cancer Center; Northeastern University

## Abstract

Type 2 Diabetes (T2D) is a condition that is often associated with obesity and defined by reduced sensitivity of PI3K signaling to insulin (insulin resistance), hyperinsulinemia and hyperglycemia. Molecular causes and early signaling events underlying insulin resistance are not well understood. Insulin activation of PI3K signaling causes mTOR dependent induction of PTEN translation, a negative regulator of PI3K signaling. We speculated that insulin resistance is due to insulin dependent induction of PTEN protein that prevent further increases in PI3K signaling. Here we show that in a diet induced model of obesity and insulin resistance, PTEN levels are increased in fat, muscle and liver tissues. Onset of hyperinsulinemia and PTEN induction in tissue is followed by hyperglycemia, hepatic steatosis and severe glucose intolerance. Treatment with a PTEN phosphatase inhibitor prevents and reverses these phenotypes, whereas an mTORC1 kinase inhibitor reverses all but the hepatic steatosis. These data suggest that induction of PTEN by increasing levels of insulin elevates feedback inhibition of the pathway to a point where downstream PI3K signaling is reduced and hyperglycemia ensues. PTEN induction is thus necessary for insulin resistance and the type 2 diabetes phenotype and a potential therapeutic target.

## Introduction

An increase in sedentary lifestyle and the dietary consumption of carbohydrates and fats leads to multiple metabolic diseases such as obesity, insulin resistance and Type 2 Diabetes (T2D). T2D is a condition that is defined by insulin resistance, hyperglycemia, and hyperinsulinemia unless it occurs with beta cell failure and insulin deficiency^1^. Insulin resistance and pre-diabetes precedes T2D and is also a driver of multiple other metabolic syndromes such as metabolic dysfunction associated steatohepatitis (MASH) and atherosclerosis^2–4^. Insulin exerts its effects by binding to and activating insulin receptor (IR) signaling. Insulin resistance is defined by a requirement for increasing concentrations of insulin to activate IR-PI3K-AKT signaling in peripheral tissues like muscle, adipose and liver. This results in insufficient uptake of glucose into these tissues and increased export of glucose from the liver^5–8^. Increased caloric intake results in hyperinsulinemia that initially maintains normoglycemia but, with time, the rising levels of insulin cannot keep up and hyperglycemia ensues. Chronic hyperglycemia and hyperinsulinemia eventually results in islet cell exhaustion and apoptosis and insulin deficiency^8^.

Insulin resistance is characterized by its reduced ability to activate IR signaling^9–13^. Insulin binding to IR causes its activation and phosphorylation of insulin receptor substrates (eg. IRS1 and 2) which bind to and activate PI3 kinase and other effector proteins. PI3kinase phosphorylates its substrate PI(4, 5)diphosphate (PIP2) leading to the production of the phosphoinositide PI(3, 4, 5) triphosphate(PIP3)^9–13^. Accumulation of PIP3 leads to the activation of AKT kinases and other substrates. AKT activation drives glucose uptake and glycogen synthesis by phosphorylating its substrates AS160 and glycogen synthase, inhibiting the former and activating the latter. Phosphorylation of AS160 causes the activation of vesicular transport of GLUT4 glucose transporter, allowing the translocation of the latter to the cell membrane and enhanced uptake of glucose^14^. In the liver, AKT activation suppresses hepatic glucose production by inhibiting gluconeogenesis and glycogenolysis and stimulating glycogen synthesis^14^. It also inhibits the transcription factors required for gluconeogenesis by phosphorylating and excluding the FOXO transcription factors from the nucleus. In adipose tissue AKT stimulates glucose uptake and inhibits lipolysis. This reduces the production of non-esterified fatty acids which are substrates for gluconeogenesis in the liver thereby further reducing hepatic glucose production^14^. The importance of both PI3K and AKT activation in maintaining glucose homeostasis is demonstrated by the rapid and marked induction of hyperglycemia in humans by drugs that inhibit either enzyme. One of the major downstream effects of AKT is induction of TOR kinase activity, which in turn, induces cap-dependent translation, ribosomal biogenesis, lipid synthesis and other processes required for cell growth^15–22^.

The output of the pathway is regulated by multiple AKT and mTOR dependent inhibitory feedback loops that limit the amplitude and duration of the signal. *PTEN* is a lipid and protein phosphatase that de-phosphorylates PIP3 to generate PIP2 thereby antagonizing PI3K activity^23^. Recently we discovered that PTEN is regulated by the PI3K/mTORC1/4E-BP1 axis via cap-dependent translation^24^. Thus, insulin activation of PI3K/AKT/mTOR increases PTEN, thereby reducing insulin signaling by lowering PIP3 levels and thereby limiting AKT activation. By contrast, nutrient starvation leads to decreased PTEN expression, and insulin sensitivity, via increased PIP3 levels, and AKT activation. This led us to hypothesize that the induction of insulin levels in people on a high caloric diet increases PTEN levels and by that mechanism, reduces AKT activation and decreases sensitivity of the cell to insulin stimulation.

## Western Diet increases PTEN expression

We hypothesized that a high fat high carbohydrate diet that causes hyperinsulinemia will also increase PTEN expression in insulin sensitive tissues. We utilized a mouse model of diet induced insulin resistance and obesity^25–27^ in which C57BL/6J 6 week old male mice were fed *ad libitum* with a “western diet” in which 41% of the calories are from fat and 43% from carbohydrates or regular chow (control diet). The former simulates the modern dietary pattern in western countries characterized by high intake of processed foods rich in refined sugars, oils, and saturated fats. This diet when given to rodents mimics a variety of human metabolic syndromes including insulin resistance and obesity^28–30^.

**Figure 1:**
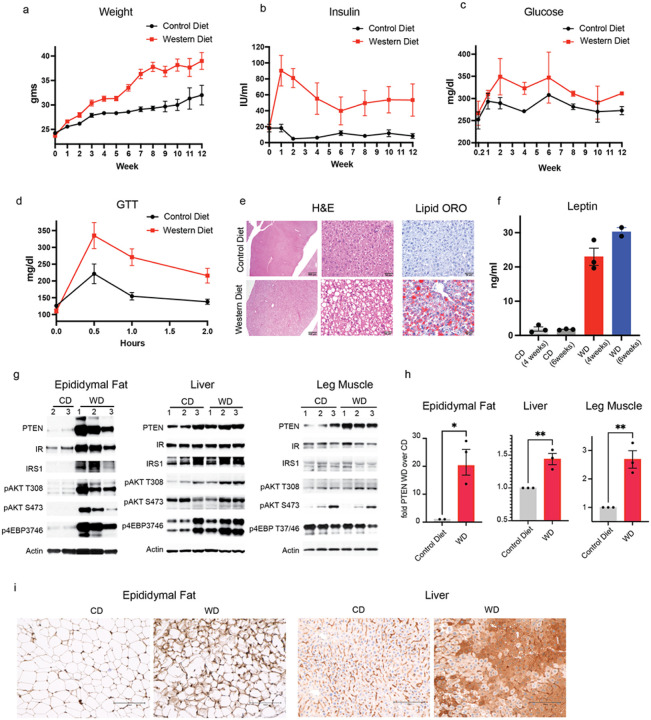
Western Diet increases PTEN expression a-c. C57BL/6J mice were either fed with regular/control (CD) or western diet (WD) for 12 weeks and the weight (n=6–10), insulin (n=3), glucose levels measured (n=3). d. After 2 weeks of control or western diet mice were fasted overnight and dosed with a bolus of glucose and their glucose levels measured for 2 hours at the indicated times for a glucose tolerance test (GTT) (n=3) e. Mice were either fed with control or western diet for 4 weeks and livers stained for H&E and Oil Red O (ORO) (n=3). Representative images shown. f. Leptin levels were measured in mice following 4 or 6 weeks of either control or western diet (n=3) g-i. g. Mice were either fed with control (CD) or western diet (WD) and proteins were extracted from eWAT, liver and muscle and analyzed by western blotting (n=3, protein from the eWAT from one control diet mouse could not be extracted) h. PTEN from g was quantified and normalized with Actin and the mean and S.E.M represented. p value was calculated using unpaired student’s t test i. eWAT and liver tissues from mice fed on CD or WD were immunostained for PTEN and representative images shown.

Mice on the western diet (WD) and control diet (CD) started gaining weight within a week and, the former, gained significantly more weight than mice on CD (65% increase in WD and 31% increase in CD after 12 weeks) (Fig. 1a). After 1 week, the serum insulin levels in mice on the western diet increased by 5-fold over that in mice on the control diet and remained increased for 12 weeks (WD over CD) (Fig. 1b, Extended Data Fig. 1a). Mice on the western diet developed hyperglycemia 2 weeks after its initiation to levels 20% over those in control mice and remained hyperglycemic over 12 weeks (Fig. 1c). Insulin sensitivity was measured using the glucose tolerance test (GTT) and a reduction in glucose tolerance began between 2 days and 1 week after western diet consumption and glucose tolerance was severely compromised by 2 weeks, coinciding with the start of hyperglycemia (Fig. 1d). Glucose intolerance increased steadily over 6 weeks of the western diet (Extended Data 1b) as demonstrated by the increase in peak glucose (30min after glucose stimulation) and adapted glucose levels (2 hours after glucose stimulation) of the GTT which remained increased by 55% and 46% respectively in mice on western diet over normal diet at the end of week 6 (Extended Data 1b, c, d). As previously reported, we found that mice on the western diet developed hepatic lipidosis and steatosis and a significant increase in leptin levels (11fold over mice on CD) within 4 weeks (Fig. 1e–f)^2,4^. Altogether these data indicates that upon high fat diet, these mice developed insulin resistance and the onset of related metabolic pathologies.

We asked whether PTEN expression was increased in insulin sensitive tissues (epididymal white adipose tissue (eWAT), muscle, and liver). PTEN expression was significantly increased in eWAT and liver tissues after 1 week of western diet and in muscle after 2 weeks (18-fold in eWAT, 1.5-fold in liver and 2.7-fold muscle) as demonstrated by immunoblotting and immunohistochemistry (Fig. 1g–i, Extended Data 1e). The increase in PTEN expression measured by immunohistochemistry in the liver was especially very striking (Fig. 1i). Phosphorylated AKT T308 and S473 also increased significantly in eWAT and AKT T308 phosphorylation increased modestly in liver and muscle (Fig. 1g). Thus, in mice on the western diet, insulin activates the expression of PTEN, a negative regulator of upstream elements of the pathway. By contrast, levels of insulin receptor and IRS1 increase in eWAT and are unchanged in the other two tissues (Fig. 1g, Extended Data 1f, g). In eWAT at 1 week, elevated insulin levels activate AKT T308 significantly, enough to maintain normoglycemia initially (Fig. 1c).

These data show that induction of insulin resistance in animals on a Western diet is accompanied by an increase in weight, serum insulin levels, insulin resistance and PTEN expression in insulin target organs.

## PTEN increases while AKT activity declines over 12 weeks in western diet

**Figure 2:**
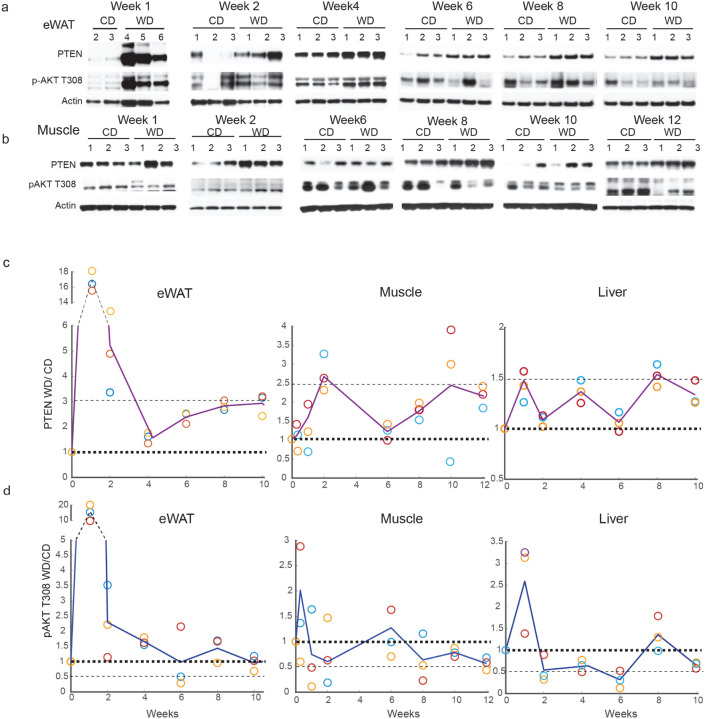
PTEN increases while AKT activity declines over 12 weeks in western diet a-b. Mice were fed regular (CD) or western diet (WD) for 12 weeks and proteins were extracted from eWAT and muscle at the indicated times and analyzed by immunoblotting for PTEN expression and AKT activity (n=3 for every time point) c-d. PTEN and pAKT from eWAT, muscle (a-b) and liver (Extended Data 2b) were quantified and normalized to actin and fold change of animals on WD over CD represented. Note; Week 1 of eWAT and liver tissues and week 2 of muscle tissue are the same as used in Figure 1 since they were a part of the same long term kinetics study that was done in Figure 2

The kinetics of changes in PTEN expression and PI3K activity on the western diet was investigated. Epididymal white adipose (eWAT), muscle and liver tissue were collected from mice that were on western or control diet as a function of time. In eWAT, PTEN and pAKT T308 increase within a week of western diet (Fig. 1g, Fig. 2a, c, d Extended Data 2a) as does Insulin (Fig. 1b). While PTEN is induced by 18fold in eWAT in mice on western diet, pAKT T308 is induced by 16-fold (Fig. 2a, c, d). Subsequently both decline and while PTEN remained upregulated to about 2–3fold for 10 weeks, pAKT declined to approximately the same levels as the control animals inspite of the high insulin production. It is likely that the PTEN declined in response to the AKT decline but remained high enough to lower AKT signaling. In the muscle tissue of mice on western diet, PTEN induction started by 1 week and peaked at 2 weeks (induced by 2.7fold over mice on control diet), remains increased by 2fold above control tissues for almost 12 weeks (Fig. 2b, c, Extended Data 2a). Phospho-AKT on the other hand, increased within 2 days after start of the western diet and continued to decline right after, its level being lower than the control tissue for the rest of 12 weeks (Fig. 2b, d, Extended Data 2a). In the liver of mice on western diet, PTEN increased within a week by 1.5fold over mice on control diet, oscillated over the next 6 weeks but remained increased after that by approximately 1.5fold until 10 weeks (Fig. 2c, Extended Data 2a-b). Phospho-AKT increased within 1 week of western diet consumption and subsequently remained reduced compared to control animals for approximately all of 10 weeks (Fig. 2d, Extended Data 2a-b).

In summary, in eWAT, within 1 week of western diet consumption there was a significant increase in AKT phosphorylation and PTEN expression that coincided with increased insulin levels (Fig. 1b, Fig. 2a, c, d). Hyperglycemia and severe glucose intolerance begun after 2weeks of western diet (Fig. 1c–d). By 10 weeks AKT phosphorylation had declined to basal levels whereas PTEN declined but remained elevated above control animal tissues. Over ten weeks, changes in serum insulin and PTEN expression in eWAT almost coincided. Thus, PTEN levels in eWAT may be regulated by insulin levels and, in turn, may decrease insulin sensitivity further. Thus, at some level PTEN induced feedback may prevent further activation of the pathway, thus leading to hyperglycemia. On the other hand, in muscle tissues, which are considered as the primary tissue of glucose uptake in response to insulin, PTEN starts increasing after a week and peaks at 2 weeks, coinciding with the onsets of hyperglycemia and severe glucose intolerance. At that time, pAKT had declined to levels 50% lower than muscle in control animals (Fig. 2b–d). Thus, unlike PTEN levels in eWAT, the PTEN levels in muscle are correlated with the onset of hyperglycemia. In the liver, the PTEN and pAKT levels increased with insulin levels (at 1 week) and pAKT levels drop below those of control animals at 2 weeks, when glucose levels rose (Fig. 2c–d).

## Inhibiting PTEN activity prevents and reverses insulin resistance

Since PTEN levels increased in insulin sensitive tissues and the increase correlated with the onset of hyperinsulinemia, hyperglycemia and glucose intolerance along with the decline of AKT phosphorylation, we asked whether inhibiting PTEN activity would affect these phenotypes. To that effect we used an inhibitor of PTEN phosphatase activity, VO-OHpic, a vanadyl compound complexed to hydroxypicolinic acid^31–33^. This complex is a non-competitive inhibitor of PTEN protein and its selectivity towards PTEN over other cysteine-based phosphatases (CBP), including protein tyrosine phosphatases, is based on exploitation of the differences in the catalytic pockets of the phosphatases. The catalytic pocket of PTEN (8Ang) is much larger than that of the other CBPs allowing this compound to bind PTEN with an IC50 of 35nM while it binds other CBPs in the uM range^31^. Its selectivity has been confirmed by its induction of AKT phosphorylation in cells with WT PTEN but not in PTEN null cells^31^. This compound has been tested in vivo in multiple pre-clinical models in which it has effectively inhibited PTEN without noticeable toxicity^31,34–36^. We confirmed that VO-OHpic induced the PI3K/AKT pathway by treatment of PIK3CA mutant BT474 and MCF7 breast cancer cells with the drug (Extended data 3a-b). Cancer cell lines with activating mutation in *PIK3CA* and wild type for *PTEN* have higher PTEN expression levels since oncogenic activation of PI3K leads to an increase in PTEN translation^24^. Upon treatment of these cells with the drug, phosphorylation of AKT, its substrate PRAS40, and mTOR substrates increased as a function of time, consistent with its inhibition of PTEN (Extended data 3a-b). In 3T3L-1 adipose cells, VOOHpic increased the duration of insulin induction of AKT, and S6K phosphorylation (Extended data 3c).

**Figure 3:**
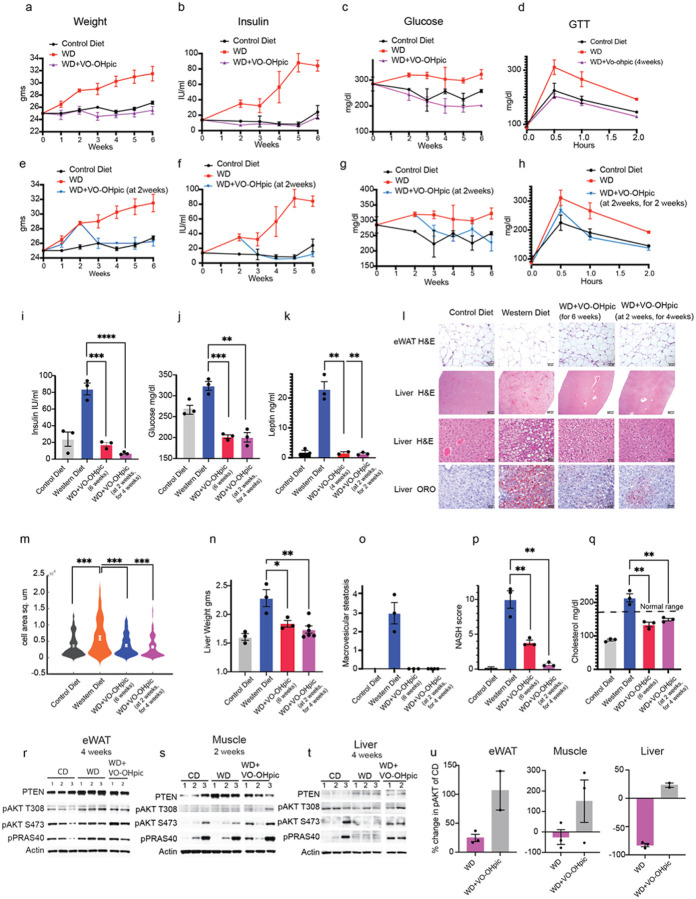
Inhibiting PTEN activity prevents and reverses insulin resistance a-c. Mice were fed on regular diet or western diet or western diet along with treatment with the PTEN inhibitor VO-OHpic (10mg/kg) once daily for 6 weeks and the weight (n=4–10), insulin, glucose (n=3) levels measured d. glucose tolerance test (GTT) (n=3) after indicated diet and treatment e-g. Mice were fed on the indicated diets for 2 weeks and then treated with VO-OHpic (10mg/kg) once daily for 4 weeks and the weight (n=4–10), insulin (n=3), glucose levels measured (n=3). The CD and WD arms of a-c and e-g are the same datasets, done together for consistency. h. glucose tolerance test (GTT) (n=3) after indicated diet and treatment. i-k. Mean and SEM of insulin (from 3b, f), glucose (from 3c, g) and leptin (n=3) of mice fed with the indicated diets and treatment. l. eWAT and livers from mice fed with the indicated diets and treatment stained for H&E and lipid OilRedO (n=3). Representative images are shown. m. Violin plot of morphometric analysis of eWAT H&E from l (n=3). n-q. The mean and SEM of the liver parameters: liver weight, macrovesicular steatosis scores, MASH scores and cholesterol levels (n=3). p value was calculated using unpaired student’s t test in i-q. r-t. Proteins from eWAT, muscle and liver of mice fed and treated with indicated conditions analyzed by immunoblotting. CD, WD same as Fig 2 since the indicated experimental arms were run on same gels together U. Quantification of percent change in pAKT over control from r-t.

Treatment of mice on the Western diet with 10mg/kg of VOOH-pic once daily led to complete prevention of weight gain over 6 weeks (Fig. 3a, Extended 3i). No toxicity of the drug was observed. Treatment with VO-OHpic completely prevented hyperinsulinemia, hyperglycemia and insulin resistance as demonstrated by glucose tolerance tests done after 4 and 6 weeks of drug treatment (Fig. 3b–d, i–j Extended Data 3g). We confirmed that the mice on western diet with and without drug treatment were consuming approximately the same amount of food (Extended Data 3d). We also confirmed that treatment with the inhibitor on control diet did not affect the weight or glucose levels of mice (Extended Data 3e-f). We asked whether inhibiting PTEN was able to reverse the phenotypes associated with insulin resistance. Mice were treated with VO-OHpic 2 weeks after initiation of the western diet, at which time hyperinsulinemia, hyperglycemia and glucose intolerance were well established (Fig. 1a–d). VO-OHpic completely reversed weight gain and hyperinsulinemia within 1 week of PTEN inhibition (Fig. 3e–f, i Extended Data 3i). Hyperglycemia and glucose intolerance were reversed after 2 weeks of inhibitor treatment and insulin sensitivity persisted on therapy and the western diet for four weeks (Fig. 3g–h, j Extended Data 3h). PTEN inhibition also prevented the development of leptin resistance and reversed the increase in leptin levels within 2 weeks of drug treatment (Fig. 3k, j). eWAT adipocytes are the primary source of leptin hormone and expand upon consumption of a high fat diet. We found that the adipocyte area per cell increased upon western diet consumption, and this was both prevented and reversed by inhibition of PTEN (Fig. 3l, m, Extended Data 3k).

PTEN inhibition also prevented the increase in liver weight upon western diet and reduced the weight upon inhibitor treatment for 4 weeks after 2 weeks of the diet exposure (Fig. 3n). Upon analysis of Oil red O (ORO) staining of liver sections, we found that lipid accumulation was substantially prevented and reversed by inhibiting PTEN at the beginning or after 2 weeks of western diet (Fig. 3l). Analysis of H&E sections, that were certified, and blind scored by a pathologist, revealed that VO-OHpic treatment completely prevented and reversed the development of macrovesicular steatosis (within 4 weeks of drug treatment for the latter) (Fig. 3o). Microvesicular steatosis was reduced by preventive treatment of VOOHpic and was reduced more by treating the animals with the drug after 2 weeks of western diet consumption (Extended Data 3l). Hepatocellular hypertrophy and lobular inflammation both were significantly reduced by preventive treatment while treatment at 2 weeks of the diet completely reversed these conditions (Extended data 3m-n). The MASH scores that are an integration of the conditions of macro- and microvesicular steatosis, hepatocellular hypertrophy and lobular inflammation, averaged around 10 for the mice on western diet, 4 for VO-OHpic treatment at the beginning of the diet and 0.5 for mice that were treated with the inhibitor after 2 weeks on western diet for 4 weeks (Fig. 3p). Other markers of liver disease or function (AST, ALT, GGT, albumin) were normal in all groups of mice (data not shown) except cholesterol which was elevated in the range of hypercholesteremia in mice on the western diet. Treatment with VO-OHpic prevented or lowered the cholesterol levels within the normal range (Fig. 3q). PTEN inhibition for 4 weeks during western diet consumption in the eWAT and liver caused an increase in AKT T308 and S473 phosphorylation (80% increase in AKT S473 in eWAT and 105% in liver of mice treated with VO-OHpic over vehicle treated mice on western diet) (Fig. 3r–s, u Extended Data 3o-p, r-s). PTEN inhibition for as little as 2 weeks in muscle tissue caused an increase of 140% in pAKT S473 in muscle (Fig. 3t, u Extended Data q, t). Phosphorylated PRAS40 and mTORC1 activity were also increased in all three tissues.

Taken together these data show that inhibition of PTEN phosphatase activity is sufficient to reverse insulin resistance and its metabolic sequelae in mice on a western diet. Insulin induction of PTEN is therefore necessary for maintenance of the phenotype. Moreover, inhibition of PTEN activity in cells in which its expression has been induced by insulin prevents the development of insulin resistance.

## Inhibiting mTORC1 prevents and reverses obesity and insulin resistance

Regulation of mTOR activation by PI3K controls the 4E-BP1 dependent translation of PTEN protein^24^. We wished to confirm that the diet dependent increase in PTEN protein in mice is sensitive to mTORC1 inhibition. mTOR phosphorylates 4E-BP and causes it to dissociate from eIF4E, thus relieving its inhibition of formation of the eIF4E initiation complex^37,38^. Stimulation of translation of capped mRNAs including PTEN mRNA ensues. In MDA-MB-468 PTEN null cells, expression of PTEN mRNA without the PTEN 5’UTR prevented the induction of PTEN expression after insulin stimulation and the duration of insulin stimulated AKT phosphorylation was increased (Extended Data 4a).

We asked whether inhibition of TORC1 kinase prevented the increase in PTEN protein by insulin stimulation. RMC-6272 is a selective inhibitor of TORC1 kinase (IC50 for mTORC1 inhibition is 0.44 nM for p4E-BP, IC50 for TORC2 inhibition of pS473 AKT is 12nM)^39^. At TORC1 selective doses this drug inhibits 4E-BP1 phosphorylation but not AKT phosphorylation, which is TORC2 dependent. RMC-6272 prevented the induction of PTEN after insulin stimulation in 3T3L1 adipocytes (Extended Data 4b).

**Figure 4:**
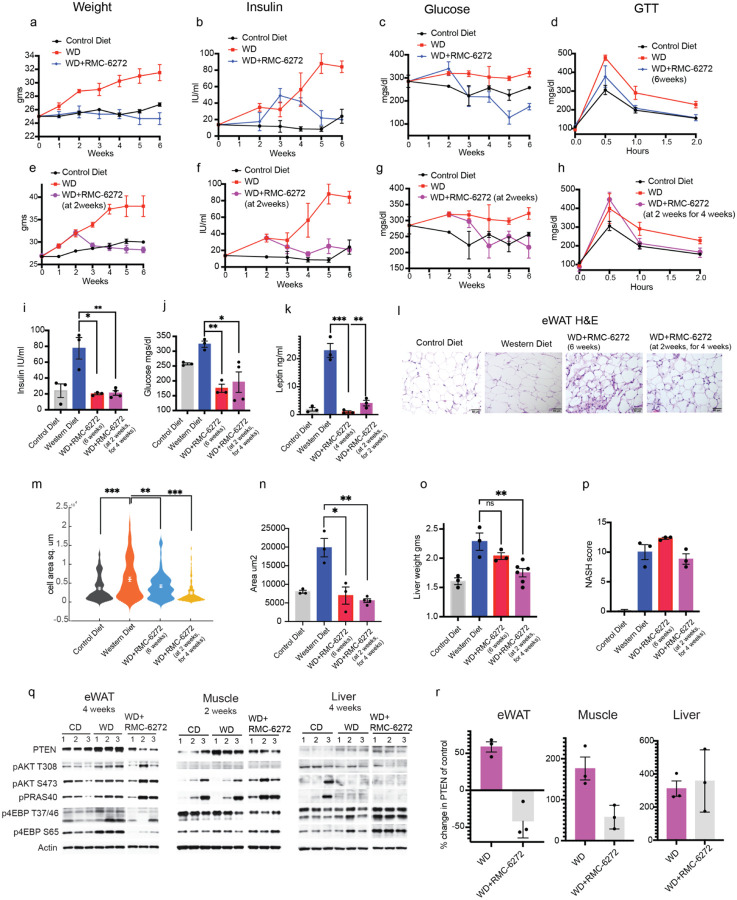
Inhibiting mTORC1 prevents and reverses obesity and insulin resistance a-d Mice were fed on regular diet or western diet or western diet along with treatment with the mTORC1 inhibitor RMC-6272 (3mg/kg) once a week for 6 weeks and the weight (n=4–10), insulin (n=3), glucose levels(n=3) and GTT measured (n=3). e-h. Mice were fed on the indicated diets for 2 weeks and then treated with RMC-6272 (3mg/kg) on western diet for 4 weeks, weight (n=4–10), insulin (n=3), glucose levels (n=3) and GTT measured. The CD and WD arms of 4a-c and 4f-g are the same datasets as 3a-c and f-g. i-k. Mean and SEM of insulin (from 4b, f) and glucose (from 4c, g) and leptin of mice fed with the indicated diet and treatment l. eWAT from mice treated with indicated conditions, stained for H&E, representative images shown (n=3). Violin plot (m) and mean and SEM (n) of morphometric analysis of l (n=3). o-p. The mean and SEM of the liver parameters weight, and MASH scores represented (n=3). p value was calculated using unpaired student’s t test in i-o. q-r. q. Proteins from eWAT, liver and muscle of mice that were fed with indicated conditions were analyzed for PTEN and PI3K pathway activation by immunoblotting. CD, WD same as Fig 2 since the indicated experimental arms were run on the same gels together r. Quantification of percent change in PTEN over control diet from q.

We tested whether RMC-6272 inhibited the increase in PTEN expression that occurs in mice on the western diet and, in doing so, prevents its induction of obesity, insulin resistance and MASH. RMC-6272 (3m/kg) was administered to mice once per week when the western diet was initiated and then once weekly. Treatment of RMC-6272 at the beginning of the diet completely prevented weight gain, reduced insulin levels after 4 weeks and glucose levels after 2 weeks to normal levels, and prevented the development of insulin resistance (Fig. 4a–d, i-j, Extended Data 4c). We confirmed that the mice in different groups approximately ate the same amount of food (Extended Data 4d, Methods) and the inhibitor alone did not induce major changes in weight or glucose levels in animals (Extended Data 4e-f).We found that weight gain, hyperinsulinemia, hyperglycemia and the development of insulin resistance were completely reversed when the mice were treated with the drug two weeks after the diet was given to them (Fig. 4e–h, i-j, Extended Data 4c). mTORC1 inhibition also prevented and reversed increases in leptin levels and adipocyte cell area in eWAT tissues (Fig. 4k–n, Extended Data 4g).

By contrast, although PTEN inhibition prevented and reversed the MASH phenotypes in mice fed on the western diet, mTORC1 inhibition did not. Liver weight, lipidosis, steatosis and overall MASH scores were not decreased by RMC-6272 (Fig. 4o–p). This result was confirmed by H&E and ORO staining of liver sections that showed no reduction in the lipid accumulation had occurred on the western diet (Extended Data 4h). Neither was the diet-induced hypercholesterolemia reduced by mTORC1 inhibition (Extended Data 4i) whereas serum liver enzymes such as serum AST, ALT, GGT as well as albumin levels were normal in all groups of mice (data not shown). To confirm that the failure to inhibit the MASH phenotype was not specific to RMC-6272, we used a different mTORC1 inhibitor, Rapamycin^40^. Rapamycin inhibited weight gain in mice on western diet and quite effectively reduced hyperinsulinemia and hyperglycemia (Extended Data 4j-l). However, H&E sections of the liver treated with Rapamycin revealed the presence of liver lipidosis (Extended Data 4m).

Lastly, we analyzed pAKT/mTORC1 activity and PTEN protein levels in the eWAT, liver and muscle tissues upon treatment with RMC-6272 or Rapamycin. Treatment of RMC-6272 or Rapamycin along with consumption of western diet for 4 weeks in the eWAT or 2 weeks in the muscle, led to a decrease in p4E-BP1 T47/46 and S65 sites and there was a significant decrease in PTEN expression accompanied by increase in pAKT and its substrate pPRAS40 (Fig. 4q–r, Extended Data 4n). In contrast, in the liver, neither RMC-6272 nor Rapamycin treatment for 4 weeks inhibited mTORC1 activity when the mice were fed with western diet, and PTEN levels were almost unchanged (Extended Data 4q-r, Extended Data 4n). Consistent with persistent PTEN overexpression, pAKT T308 phosphorylation and that of the AKT substrate remained low.

In summary, inhibiting PTEN activity in the insulin sensitive tissues inhibited the obesity, insulin resistance and MASH phenotypes whereas inhibiting mTORC1, suppressed PTEN and its metabolic effects in eWAT and muscle but not liver. The difference seems to be the effectiveness of the mTORC1inhibitors to reduce PTEN in the liver.

Insulin resistance and subsequent type 2 diabetes are complex phenomena for which there is currently no unitary explanation^41^. No single primary event that affects the insulin signaling pathway and explains multiple features of the diseases has been found, although in single cases and in families with inherited disorders of glucose homeostasis it has been shown to be due to single gain or loss of function mutations that affect signaling (eg insulin receptor, IRS proteins, TSC2, AKT2)^42–44^. Various phenomena that are part of the syndrome (increased lipolysis in fat cells, increased gluconeogenesis and glycogenesis in liver) have been known to cause other aspects of the phenotype^14^. Changes in neural and endocrine regulation of appetite have been shown to be associated with the syndrome but beg the question of how increased food intake initiates the problem^2,4^.

We show here a relatively simple mechanism that explains insulin resistance with attendant effects on white adipose, liver, and muscle cells that explain many of the features of type two diabetes. It is based on a recent finding that the translation of PTEN, a potent downstream negative regulator of insulin signaling, is mTOR dependent^24^. Activation of the insulin/PI3K/AKT/mTOR pathway is therefore buffered by induction of PTEN expression. Similarly, nutrient deprivation or reduction in insulin signaling causes a fall in PTEN expression, allowing of some level of AKT signaling. Hence, PTEN is a powerful feedback regulator of insulin signaling that is predicted to play an important role in metabolic homeostasis.

This model generated the hypothesis that hyperactivation of insulin could lead to an overshoot of PTEN expression and thus insulin resistance. We show here that this is the case in mice on a high fat and carbohydrate diet. After initiation of the diet, insulin levels increase and PTEN expression increases in white adipose tissue, muscle and liver. This is followed by weight gain, hyperglycemia, insulin resistance, hepatic lipidosis, and leptin resistance. In support of our hypothesis, each of these is prevented or reversed by administration of a selective inhibitor of the PTEN lipid phosphatase. In confirmation, patients with Cowden’s Syndrome and monogenic PTEN mutations that lead to its haploinsufficiency are more sensitive to insulin action and protected from insulin resistance^45,46^. Caloric restriction is also expected to inhibit mTOR and thereby inhibit PTEN. This may explain in part why it is effective in treating insulin resistance and T2D^47^. Conversely a cohort of T2D patients from Japan were found to harbor mutations in the 5’UTR region of PTEN that led to an increase in its protein translation^48^. It has also been reported that 4E-BP1 and 4E-BP2 double knockout mice have increased sensitivity to obesity and insulin resistance whereas overexpressing 4E-BP1 makes mice resistant to the phenotypes^49,50^. This could be explained by increased translation of PTEN protein in 4E-BP knockout mice and its decrease in the overexpression models.

PTEN inactivates AKT signaling by dephosphorylating PIP3^23^. PTEN is both a lipid and protein phosphatase and was previously shown to dephosphorylate the tyrosine 612 residue on IRS1 leading to IRS1 and then AKT activations^51^. Thus, induction of PTEN by the insulin pathway may cause feedback inhibition of the pathway by two mechanisms. This model may explain the loss of IRS1 activity in mice with insulin resistance and in women diagnosed with gestational diabetes that has been reported before^52–54^.

This paper reports a potential mechanism for development of insulin resistance and diabetes, but it may also have therapeutic implications. In animals treated with a selective PTEN inhibitor, insulin resistance and some of its key biological consequences are prevented and reversed, suggesting the potential use of such a drug in patients. We do not know the long term consequences of taking a PTEN inhibitor. PTEN is a tumor suppressor gene and loss of a single copy may be haploinsufficient. It may be possible to determine doses that inhibit elevated activity of the protein but do not cause inhibition below physiologic levels. This has not yet been tried. Another possibility is the use of mTOR inhibitors which reduce PTEN translation and expression. However, although we found that mTORC1 selective inhibitors reverse obesity and insulin resistance in mice on the Western diet they do not reverse MASH. This is associated with desensitization of liver mTORC1 to these drugs. Moreover, mTORC1 inhibition with Rapalogs have been observed to induce paradoxical insulin resistance^55^.

## Materials and Methods

### Animal studies-weight measurements and blood parameters

For the western diet induced obesity and insulin resistance phenotypes, C57BL/6J mice at 8 weeks of age were placed on either a standard laboratory rodent chow or western diet (D12079B, Research Diets) and allowed to eat ad *libitum* and their weights measured as indicated. Mouse blood serum was collected and measured for glucose, cholesterol and liver function tests using chemical analyzers and insulin and leptin using ELISA at the indicated times.

### Glucose tolerance test

Mice were fasted for 6 hours, and glucose (2g/kg) was intraperitoneally injected into the mice and blood collected from the tail vein and glucose concentrations were determined at the 30minutes, 1hr and 2hrs.

### Tissue collection and western blotting

Epididymal white adipose tissue, liver, leg and arm muscles were collected from mice at the end of each time point (as indicated) and flash frozen. They were homogenized in SDS lysis buffer (50mM Tris-HCL pH 7.4, 10% Glycerol, 2% SDS) and boiled at 95 C for five minutes. Lysates were then briefly sonicated, boiled again for 5 minutes, before clearing by centrifugation at 14,000rpm for 10 minutes at room temperature. The supernatant was collected, and protein concentration was determined using the BCA kit (Pierce) per manufacturer’s instructions. Protein samples were diluted in SDS sample buffer (final concentration: 62.5mM TrisHCL pH 6.8, 2% SDS, 10% Glycerol, 15.5mg/mL DTT, 0.02mg/mL Bromophenol blue). 25–50 mg of protein was loaded onto each lane of a 4–12% BisTris mini gel or midi gel (Invitrogen) for immunoblotting. Transfer was onto nitrocellulose membranes (0.2 mm, GE Health Care) before blocking for 1h at room temperature and incubating with primary antibodies of the indicated protein targets overnight at 4 C. Membranes were incubated with secondary rabbit antibody (Sigma) or secondary mouse antibody (GE Health Care) for 1h at room temperature. Blots were developed in Perkin-Elmer’s Western Lightning ECL or Millipore’s Immobilon HRP reagents per manufacturer’s instructions.

### Cell lines and Antibodies and drugs

BT474, MCF7, MDA-MB-468 cells were acquired from ATCC and cultured in DMEM-F12 and 3T3L-1 was cultured in DMEM. All cell lines were supplemented with 10% Fetal Bovine serum (FBS) and 1% penicillin and streptomycin and 4mM Glutamine.

Antibodies used are PTEN (CST #9559), pAKT T308 (CST #2965), pAKT S473 (CST #4060), pPRAS40 (CST #2997), p4E-BP1 T37/46 (CST#2855), p4E-BPs S65 (CST #9451), IRS1 (CST# 2382), IR-ß (CST #3025), ß-Actin (CST #4970)

### Histological analysis

Representative sections of the liver, pancreas, brain, epididymal adipose tissue, retroperitoneal white adipose tissue, skeletal muscle from forelimbs, and skeletal muscle from the hindlimbs were fixed in 10% neutral-buffered formalin, processed in alcohol and xylene, embedded in paraffin, sectioned (5-μm-thick) and stained with hematoxylin and eosin. Oil red O staining was performed on formalin fixed, OCT-embedded frozen sections (5-μm-thick) of liver. For histopathological analysis, hematoxylin–eosin-stained or ORO-stained tissue specimens were evaluated by a board-certified veterinary pathologist (S.E.C.). Liver sections were evaluated and scored, using a semiquantitative histopathology scoring system, with slight modifications, for mouse model of metabolic dysfunction associated fatty liver disease^56^. Briefly, macrovesicular steatosis, microvesicular steatosis and hepatocellular hypertrophy were separately scored, and the extent and severity of the lesions were graded, into the following categories: 0 (< 5%), 1 (5–10%), 2 (10–25%), 3 (25–75%) and 4 (> 75%). Inflammation was evaluated by counting the number of inflammatory foci per five 100x fields using the following categories: normal (< 0.5 foci), minimal (0.5–1.0 foci), mild (1.0–2.0 foci), moderate (2.0–5.0 foci), severe (> 5.0 foci). An Olympus BX45 light microscope was used to capture images with a DP26 camera using cellSens. Dimension software (v1.16).

### Immunohistochemistry

Immunolabeling of PTEN in liver and epididymal white adipose sections was performed at the MSK Biobank and Pathology Core facility. Formalin-fixed, paraffin-embedded sections were stained using an automated staining platform. Briefly, following deparaffinization and heat-induced epitope retrieval, the primary antibody against PTEN (1:200, Cat. No 9559, clone 138G6, Cell Signaling Technologies)^57^.

### Morphometric analysis of eWAT

Cell size distribution in hematoxylin-eosin (H&E)-stained sections of epididymal white adipose tissue was analyzed from triplicates of 40X images per group and cell size was quantified using Adiposoft Software (Image J)^58^

### Treatment with PTEN inhibitor VO-OHpic

VO-OHpic was suspended in 2% DMSO, 40% PEG 300, 5% Tween-80, ddH2O and administered intraperitonially at a dose of 10mg/kg, every day, once a day. This was done either at the same time as the start of the western diet in mice for 6 weeks or after 2 weeks of western diet feeding for 4 weeks

### Treatment with mTORC1 inhibitor RMC-6272 or Rapamycin

RMC-6272 was suspended in 1:1 (v/w) Transcutol/Solutol HS 15 and administered intraperitonially at a dose of 3mg/kg, once a week. This was done either at the same time as the start of the western diet in mice for 6 weeks or after 2 weeks of western diet feeding for 4 weeks. Rapamycin was dissolved in 100% DMSO and administered intraperitonially at a dose of 10mg/kg, three times a week along with western diet.
